# Multi-sensor geospatial modelling to address complex mangrove dieback: misattribution of chemical stressors versus physical impact

**DOI:** 10.1007/s10661-026-15666-7

**Published:** 2026-07-07

**Authors:** Jade Farrugia

**Affiliations:** 1 School of the Environment, The University of Queensland, Brisbane, QLD Australia; 2 The Oceans Need Us, Bribie Island, Australia

**Keywords:** Mangrove dieback, Forensic ecology, Sentinel-2, Hydrological trap, Boambee Creek, Restoration

## Abstract

Accurately identifying the causes of mangrove dieback is essential for global coastal management, yet visually similar dieback events can result in incorrect attribution of causes. A significant mangrove dieback in Boambee Creek Estuary (2021) was initially linked to chemical contamination from nearby Coffs Harbour Airport. This study uses a forensic geospatial reconstruction to test the validity of this toxicity hypothesis against a physical-hydrological alternative. Scaling up from localised, site-specific field observations, we used multi-sensor satellite telemetry (Sentinel-2), LiDAR-based geomorphic modelling and ERA5 climatological reanalysis to assess the ecosystem at the landscape scale. The investigation pinpoints a statistically extreme hailstorm on 20 October 2021 as the trigger event, representing a > 1-in-100-year anomaly (*Z* = 4.10σ). Time-series diagnostics confirm an immediate structural collapse (*p* < 0.001) coinciding with the storm, ruling out the signature of gradual chemical ageing. Mortality followed a Death Curve, where the likelihood of death neared 100% at elevations below 1.5 m AHD (*p* < 10^–8^) within stagnant topographic basins (< 2° slope). Hydrological routing shows that the main runoff from the airport flows directly into the remaining forest, creating a Runoff Paradox that statistically discredits the chemical vector hypothesis. We conclude that the dieback is best explained by a compound disturbance framework, primarily triggered by a hydrological trap. Sudden physical defoliation halted canopy transpiration, causing rapid soil anoxia and root drowning in geomorphically unstable basins. Secondary chemical stressors may inhibit long-term recovery. Future management should focus on restoring hydrological connectivity rather than chemical remediation. This study highlights the vital need to incorporate landscape-scale multi-sensor remote sensing (Optical, Radar and LiDAR) for validating localised field sampling and accurately diagnosing heterogeneous dieback events worldwide.

## Introduction

### Global context: mangrove resilience and vulnerability

Mangrove ecosystems are among the most carbon-rich and biologically active environments on Earth, providing vital services from protecting coastlines to supporting fisheries (Donato et al., 2011). Despite their adaptations to the fluctuating intertidal zones, like aerial root systems (pneumatophores) for gas exchange and salt-exclusion mechanisms, mangroves operate near their physiological limits (Lovelock et al., 2015). They are increasingly threatened by a combination of human-related stressors and worsening climate conditions. Understanding what causes mangrove death is essential for conservation efforts. Ecological disruptions are usually classified as either pulse events (sudden, short-term shocks such as cyclones, hailstorms or tsunamis) or press events (ongoing, long-term stressors like sea-level rise, chemical pollution or changes in hydrology) (Duke et al., 2017). Although mangroves are evolutionarily adapted to recover from pulse events through epicormic resprouting and seedling recruitment, the combination of a physical pulse and persistent hydrological pressure can cause catastrophic ecosystem failure, known as peat collapse or drowning (Cahoon et al., 2003). Differentiating these drivers is often difficult because of the diverse nature of estuarine environments. A single mortality event may seem to result from a visible, immediate cause (such as pollution sources), while the underlying reason (such as geomorphic vulnerability) remains hidden. This study investigates one such complex mortality event in the Boambee Creek Estuary, New South Wales, where competing ideas, chemical toxicity versus physical-hydrological failure, have significant implications for management.

### The Boambee Creek mortality event

In late 2021, a significant dieback event occurred in the mangrove forests of Boambee Creek, which includes *Avicennia marina* and *Aegiceras corniculatum*, near the Coffs Harbour Regional Airport. The dieback was patchy, marked by a clear Dead Zone with no canopy, and a nearby Survivor Zone that showed resilience despite being in the same climate zone. The event happened alongside a severe weather event on 20 October 2021, featuring heavy rain and hailstorms (Bureau of Meteorology, 2021). The ongoing dieback and failure to recover in some areas led to investigations into human-related causes. Initial studies indicated chemical contamination from the nearby airport was the main cause (Benkendorff et al., 2025). An alternative hypothesis proposed in this study is that acute physical storm damage can produce symptoms visually similar to those of chemical stress, leading to the misattribution of toxicity.

### The chemical toxicity hypothesis: a critical review

A recent study by Benkendorff et al. (2025) examined this event and concluded that chemical contamination was the main cause of death. Their study suggested that hydrocarbon deposits from the nearby airport runway and flight path, possibly from fuel dumping or unburned Jet A1 residue, built up in the sediment, causing phytotoxicity. This finding was largely based on the detection of Total Petroleum Hydrocarbons (TPH) in sediment samples taken after the deaths. Although Benkendorff et al. (2025) provided important biochemical data, the idea that chemical toxicity caused the mortality faces several methodological and theoretical issues that need re-examination.

A key limitation of the previous analysis was its dependence on a binary comparison between two single locations (*N* = 2). In complex estuarine environments, a sample size of two is inadequate to distinguish site-specific geomorphic features, such as elevation and drainage, from larger landscape stressors. Without a wider spatial assessment, it is impossible to determine whether the impact site failed due to pollution or simply because it functioned as a topographic sink. Drawing landscape-scale conclusions from such a small sample carries a high risk of selection bias, particularly Pseudoreplication, which can easily cause confusion between correlation and causation (Hurlbert, 1984).

Detecting hydrocarbons in dead mangrove stands does not necessarily prove they caused the death. Mangrove sediments are known to trap lipophilic contaminants (Tam & Wong, 2000), and *Avicennia marina* is well known for tolerating moderate hydrocarbon levels (Wardrop et al., 1987). The Association is not Causation fallacy is especially relevant here; the high volatility of low-molecular-weight hydrocarbons in subtropical estuaries usually prevents them from persisting long in the water column. Linking mortality to acute water-column toxicity needs evidence of a concentration gradient matching the pattern of death, rather than just detecting a point source after the event.

The chemical hypothesis assumes that runoff from the airport is the cause of toxicity. This creates a hydrological paradox. If the runway runoff is the source of the toxin, hydrological flow models suggest that the Survivor site, which is also near the runway catchment, should have been equally exposed. If the Control site receives the same or more runoff than the impact site but remains unaffected, the idea of acute water-column toxicity is logically contradicted.

### The physical-hydrological hypothesis

An alternative hypothesis proposed here is that the mortality resulted from a biophysical failure arising from the interaction between Acute Physical Trauma and Hydrological Incompetence. Mangroves depend on atmospheric oxygen diffusing through lenticels on their aerial roots to oxygenate their subterranean root systems (Scholander et al., 1955). This process is driven by transpiration, which creates a negative pressure gradient known as biological pumping (Steudle, 2001). Severe hailstorms can cause complete defoliation, instantly stopping transpiration. If this defoliation occurs in a basin that cannot drain (a hydrological trap), the loss of biological pumping, combined with static floodwaters, leads to rapid soil anoxia (McKee, 1993). Under these conditions, root drowning can occur within days to weeks, causing irreversible mortality regardless of chemical presence. This mechanism indicates that Elevation and Slope, rather than proximity to a flight path, are the primary factors influencing survival. Trees in low-lying basins drown after defoliation, while trees on slopes that drain survive.

### Objectives of the forensic reconstruction

To settle this debate, this study scales up from localised field sampling to a landscape-scale forensic reconstruction. Recent advances demonstrate that high-frequency satellite telemetry provides an unprecedented, landscape-scale advantage for monitoring dynamic mangrove ecosystems, overcoming the spatial and temporal limitations of traditional field sampling (Ghorbanian et al., 2021; Wang et al., 2019). By combining multi-sensor satellite telemetry (Sentinel-1 SAR, Sentinel-2 MSI), climatological reanalysis (ERA5) and high-resolution LiDAR hydro-topographic modelling, we aim to identify the physical and chemical drivers of the Boambee Creek dieback.

This study pursues three main research goals. First, we assess the meteorological trigger to determine whether the event on 20 October 2021 was a black-swan shock capable of causing physical structural damage or simply a normal seasonal variation. Second, we use spatiotemporal diagnostics with Before-After-Control-Impact (BACI) analysis to establish if the dieback was instant, indicating physical trauma, or gradual, suggesting chronic chemical stress. Third, we examine the mechanism of mortality by modelling the statistical relationship between mortality, elevation and drainage slope across the entire estuary. This includes explicitly mapping the airport runoff pathways to test the validity of the toxicity vector. This forensic approach offers a rigorous, statistically significant framework (*p* < 0.001) for identifying the true cause of the ecosystem collapse.

## Methodology

### Study area

**Fig. 1:**
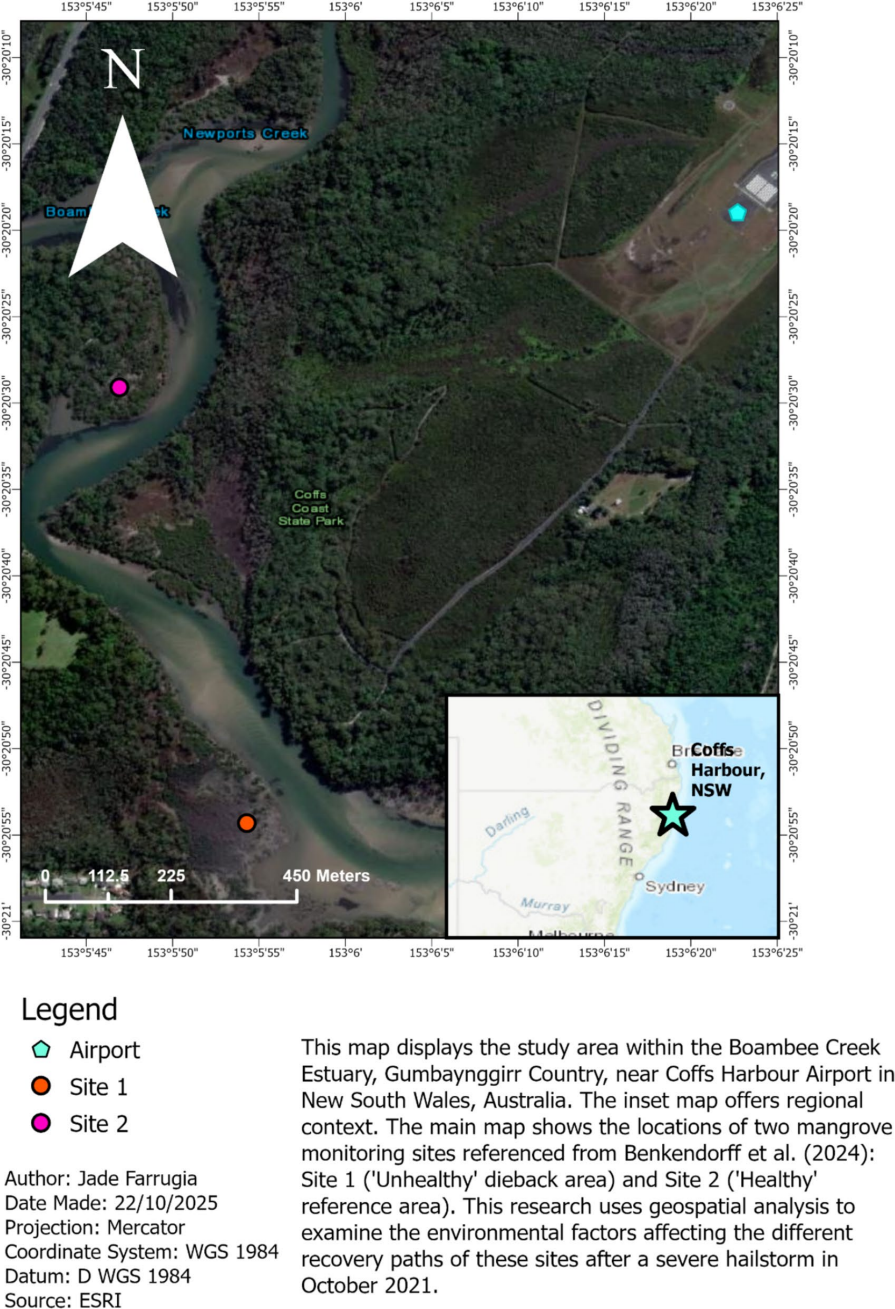
Location of the study area in Boambee Creek Estuary, Gumbaynggirr Country, near Coffs Harbour Airport, NSW, Australia. The map shows the unhealthy impact site (site 1, orange) and the healthy control site (site 2, pink) referenced by Benkendorff et al. (2025). The airport runway’s close proximity to the estuary in the north supports the chemical contamination hypothesis tested in this study

The study examines the Boambee Creek Estuary (30.35°S, 153.10°E), a subtropical estuarine system on the mid-north coast of New South Wales, Australia. The system is mainly composed of Grey Mangrove (*Avicennia marina*) and River Mangrove (*Aegiceras corniculatum*) (Duke, 2006). The site is bordered to the north by Coffs Harbour Regional Airport and experiences mixed semi-diurnal tides. To improve statistical accuracy, the Area of Interest (AOI) was defined not only by the two specific sites noted in previous research (Impact and Control) (Benkendorff et al., 2025) but also to encompass the entire connected mangrove area within the catchment, enabling stratified random sampling of over 1300 pixels (Fig. 1).

### Meteorological forensics (ERA5)

To objectively assess the severity of the alleged trigger event (20 October 2021), we used ECMWF ERA5-Land reanalysis data (Hersbach et al., 2020). We extracted hourly precipitation and wind gust vectors to characterise the storm’s convective profile. A 20-year precipitation baseline (2000 –2021) was established to determine the climatological mean (μ) and standard deviation (σ) for the area. The storm’s intensity was then standardised to a Z-Score (*Z* = (*x *− μ)/σ). This measure helps identify how rare the event is statistically, differentiating between typical seasonal rainfall and extreme shock events that can cause physical structural damage.

### Spatiotemporal reconstruction (BACI)

We used a Before-After-Control-Impact (BACI) design (Underwood, 1994) with satellite telemetry to reconstruct the collapse timeline. Sentinel-2 (Optical) imagery (Drusch et al., 2012) was accessed via Google Earth Engine (Gorelick et al., 2017). To capture rapid phenological changes, we extracted the spatial mean of the Normalised Difference Vegetation Index (NDVI) (Tucker, 1979) and the Normalised Difference Moisture Index (NDMI) (Gao, 1996) for both study sites across all available cloud-free Sentinel-2 passes from 2020 to 2024. Cloud masking was applied using the QA60 band (cloud and cirrus bitmasks) to maintain radiometric integrity. A Welch’s *t*-test was conducted on the difference in vegetation indices (Δ_*NDVI*_) before and after the storm to check for structural breaks in the time series.

We examined the biophysical connection of the canopy. In healthy mangrove ecosystems, canopy greenness (NDVI) and leaf moisture (NDMI) are closely linked traits (Ceccato et al., 2001). We determined the Pearson correlation coefficient (*r*) between these two indices for both the pre-storm and post-storm periods. A notable decoupling of these indices was seen as a sign of severe defoliation and hydraulic failure.

### Hydro-topographic auditing

To test the hydrological trap hypothesis, a 1-m LiDAR-derived Digital Elevation Model (DEM) (NSW Government Spatial Services, 2025) was used to extract geomorphic variables for each estuary pixel.

We conducted an estuary-wide logistic regression to model the likelihood of mortality (*P*_*death*_) as a function of elevation. This involved sampling *N* = 1306 pixels across the mangrove area, classifying them as Dead (dNDVI < − 0.15) or Alive, and fitting a sigmoid probability curve (Zuur et al., 2009). This method advances beyond anecdotal site comparisons to establish a statistical rule at the landscape scale.

Geomorphic drainage potential was evaluated by calculating the slope (degrees) and topographic curvature. Hydrological routing was performed in ArcGIS Pro (Esri, 2025) using the D8 flow direction algorithm (O’Callaghan & Mark, 1984) to map surface runoff pathways from the airport runway. This enabled verification that the Impact site was the primary recipient of runway runoff, thereby testing the validity of the chemical vector hypothesis.

### Evaluation of classification accuracy

To validate the spatial extent of the damage, a change detection map (dNDVI) was created by subtracting the post-storm median (Nov 2021–Jan 2022) from the pre-storm median (Jul–Sep 2021). The accuracy of this classification was confirmed using a pixel-level confusion matrix (*N* = 114) (Congalton, 1991). Ground truth labels were obtained from high-resolution aerial imagery and the known status of the impact and control sites. Sensitivity, specificity and overall accuracy were calculated to ensure that the identified Dead Zone represented a distinct population rather than random noise.

### Statistical Analysis

All statistical analyses were conducted in Python using the pandas, scipy.stats, sklearn (Pedregosa et al., 2011) and statsmodels libraries. Differences in mean elevation and slope between the Impact and Control sites were assessed with independent t-tests. The significance of divergence in the time series was assessed with Welch’s t-test for unequal variances. The relationship between elevation and mortality probability was examined with logistic regression and Point-Biserial correlation, while post-storm recovery trajectories were modelled with locally weighted scatterplot smoothing (LOWESS). All tests were considered significant at *⍺* = 0.05, with high-significance thresholds set at *p* < 0.001.

## Results

### The meteorological trigger: a statistical Black Swan

**Fig. 2:**
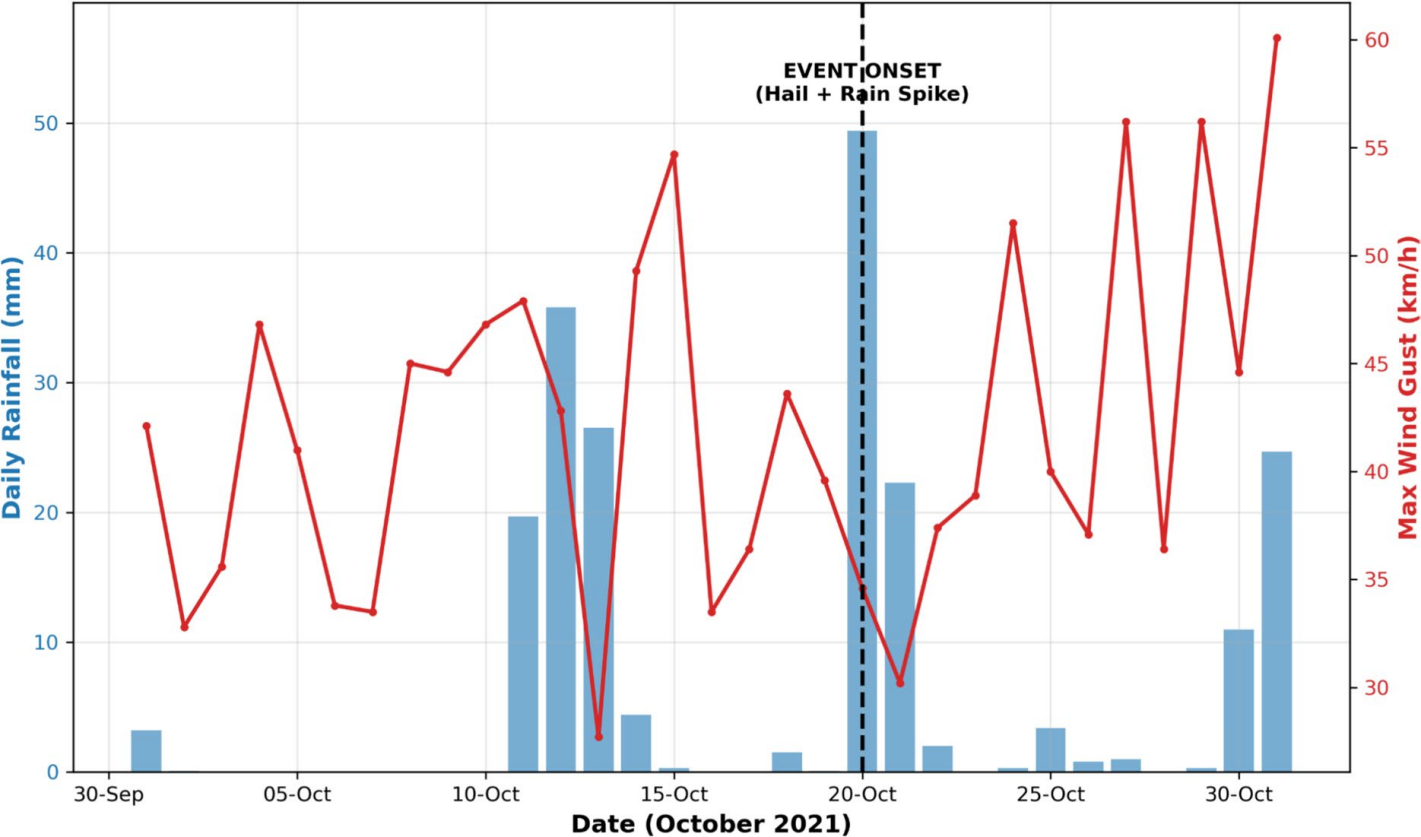
Meteorological signature of the Black Swan event on 20 October 2021. ERA5 reanalysis data show a statistically extreme precipitation anomaly (*Z* > 4.10σ) coinciding with the onset of dieback. Note the clear absence of high wind gusts (< 20 km/h) during the precipitation spike (Blue), confirming the event was not a wind-driven cyclone but rather a vertical convective downburst (hail/rain) event. This aligns with the observed pattern of standing dead damage

To validate the hypothesis of acute physical trauma, the meteorological conditions of the alleged trigger event (20 October 2021) were analysed against a 20-year climatological baseline. The ERA5 reanalysis data (Hersbach et al., 2020) confirmed that the Boambee Creek catchment experienced a statistically extreme hydrometeorological anomaly. The storm event registered a precipitation Z-Score of 4.10σ relative to the historical mean (Table 1). In standard normal distribution terms, a deviation of this magnitude corresponds to a probability of occurrence of less than 0.01%, classifying the storm as a shock event exceeding the 1-in-100-year recurrence interval. This event was characterised by low surface wind speeds (< 20 km/h) but extreme vertical precipitation intensity (Fig. 2). This sets the event apart from a wind-driven cyclonic disturbance. The combination of low horizontal wind shear and high-intensity precipitation aligns with a quasi-stationary convective cell capable of producing large-diameter hail. This explains the specific damage pattern observed: trees were not uprooted by wind, but rather defoliated and debarked on site by the vertical kinetic energy of falling hydrometeors.

**Table 1:** Summary of the forensic meteorological and geomorphic review for the Boambee Creek mortality event. Meteorological data is sourced from ERA5 reanalysis (2000–2021 baseline). Geomorphic data is obtained from a 1-m LiDAR DEM analysis

Parameter	Value	Classification/interpretation
Meteorological trigger (ERA5)		
Event date	20 October 2021	Hailstorm/severe storm
Precipitation Z-score	4.10σ	Extreme shock event (> 1-in-100-year recurrence)
Geomorphic drainage (LiDAR)		
Impact site (site 1) slope	1.99°	Flat/stagnant basin (hydrological trap)
Control site (site 2) slope	5.09°	Drainage slope (effective de-watering)
Drainage ratio	2.6:1	Site 2 is 2.6 × steeper than site 1

### Spatiotemporal forensics: instantaneous ecosystem collapse

The reconstruction of the ecosystem’s physiological trajectory using Sentinel-2 telemetry uncovers a clear temporal signature of collapse. The Before-After-Control-Impact (BACI) analysis shows that before the storm event, the Impact Site (Site 1) and the Control Site (Site 2) had synchronised phenological cycles, with no significant statistical difference in mean Normalised Difference Vegetation Index (NDVI). This synchrony suggests that both sites faced similar environmental conditions and had comparable baseline health.

**Fig. 3:**
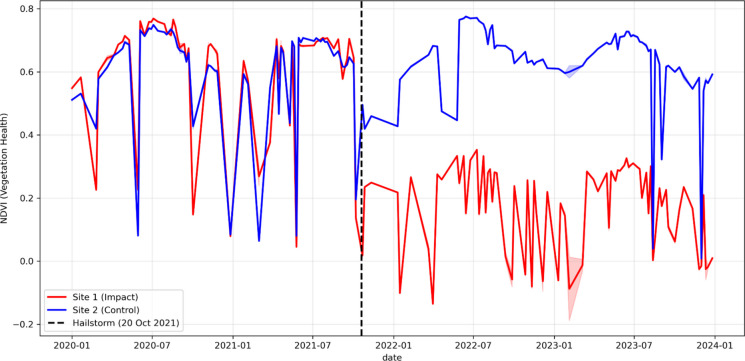
Spatiotemporal reconstruction of ecosystem health (2020–2024) using the spatial mean of Sentinel-2 NDVI across all available cloud-free Sentinel-2 passes for both sites. The vertical dashed line indicates the hailstorm event (20 Oct 2021). Before the event, the impact site (red) and control site (blue) showed synchronous phenology. During the storm, site 1 experienced an immediate structural collapse. A Welch’s *t*-test confirms the post-storm divergence is highly significant (*p* < 0.001), refuting the hypothesis of gradual chemical senescence

Coincident with the storm date, the time series shows an immediate structural break. The NDVI at site 1 plummeted sharply, diverging from the Control site’s trajectory straight after the event (Fig. 3). A Welch’s *t*-test on the pre- and post-storm mean differences (Δ_*NDVI*_) confirms this divergence is highly statistically significant (*t* = 22.88, *p* < 0.001). The rapid occurrence of this break, within a single satellite capture interval, rules out the hypothesis of chemical senescence. Phytotoxicity caused by soil hydrocarbons usually appears as a slow physiological decline (chlorosis) over months or years. The sudden, steep drop in vegetation indices is a clear spectral sign of acute physical defoliation.

**Fig. 4:**
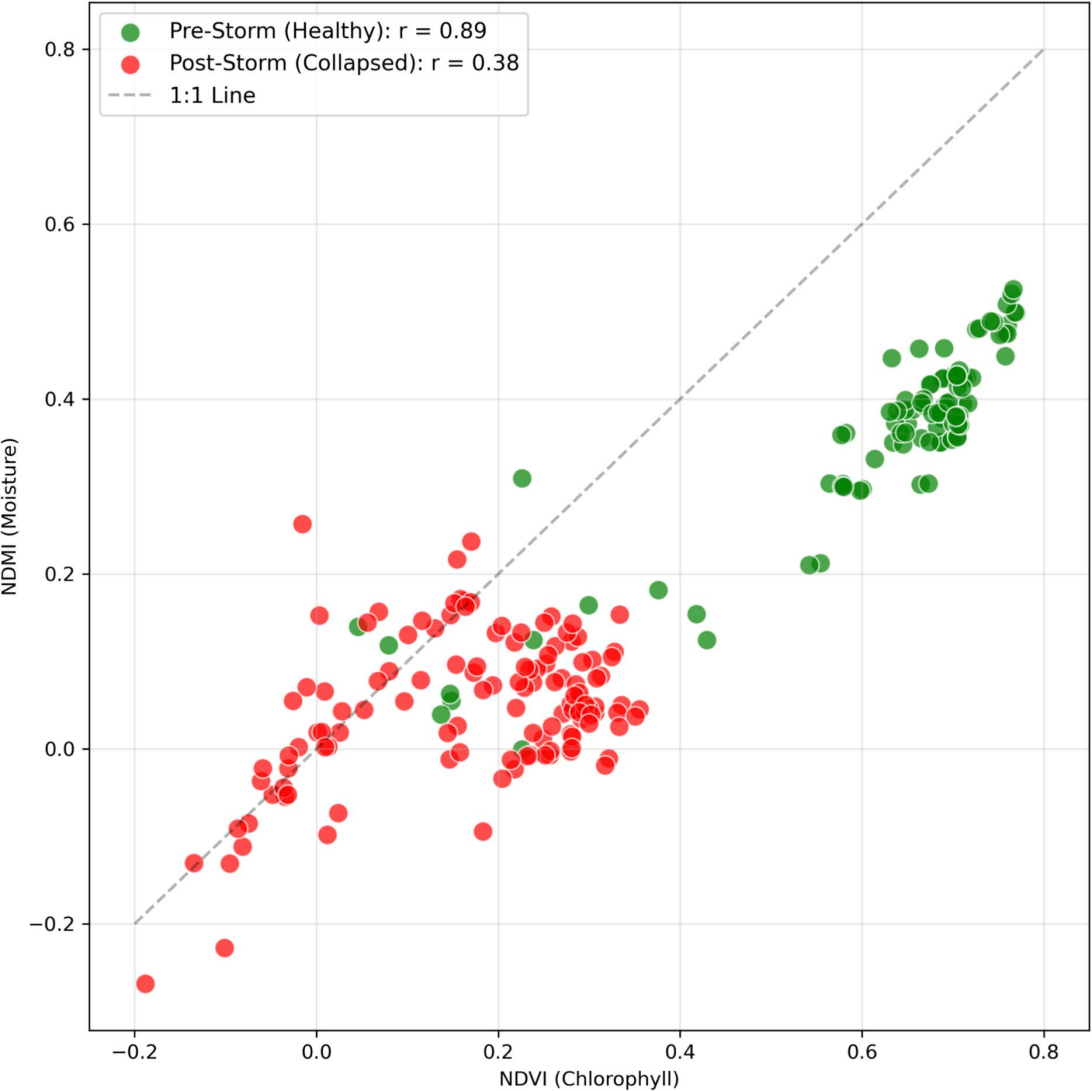
Biophysical state space analysis illustrating physiological decoupling. Pre-storm conditions (green) show a strong coupling (*r* = 0.89) between canopy greenness (NDVI) and leaf moisture (NDMI), indicating a regulated hydraulic system. Post-storm conditions (red) at site 1 reveal chaotic decoupling (*r* = 0.38) and state collapse, a diagnostic marker of acute defoliation and xylem failure rather than chronic toxicity

This structural collapse was further supported by an analysis of the biophysical state space. In the pre-storm phase, the ecosystem maintained a tightly linked relationship between canopy greenness (NDVI) and leaf moisture content (NDMI), with a Pearson correlation coefficient of *r* = 0.89. This strong link indicates a functional hydraulic system where chlorophyll content increases linearly with turgor pressure. In the post-storm phase, this relationship decoupled considerably (*r* = 0.38) (Fig. 4), suggesting a chaotic breakdown of physiological regulation consistent with the severing of the hydraulic continuum due to extensive leaf loss and subsequent xylem cavitation.

### Spatial extent and classification accuracy

**Fig. 5:**
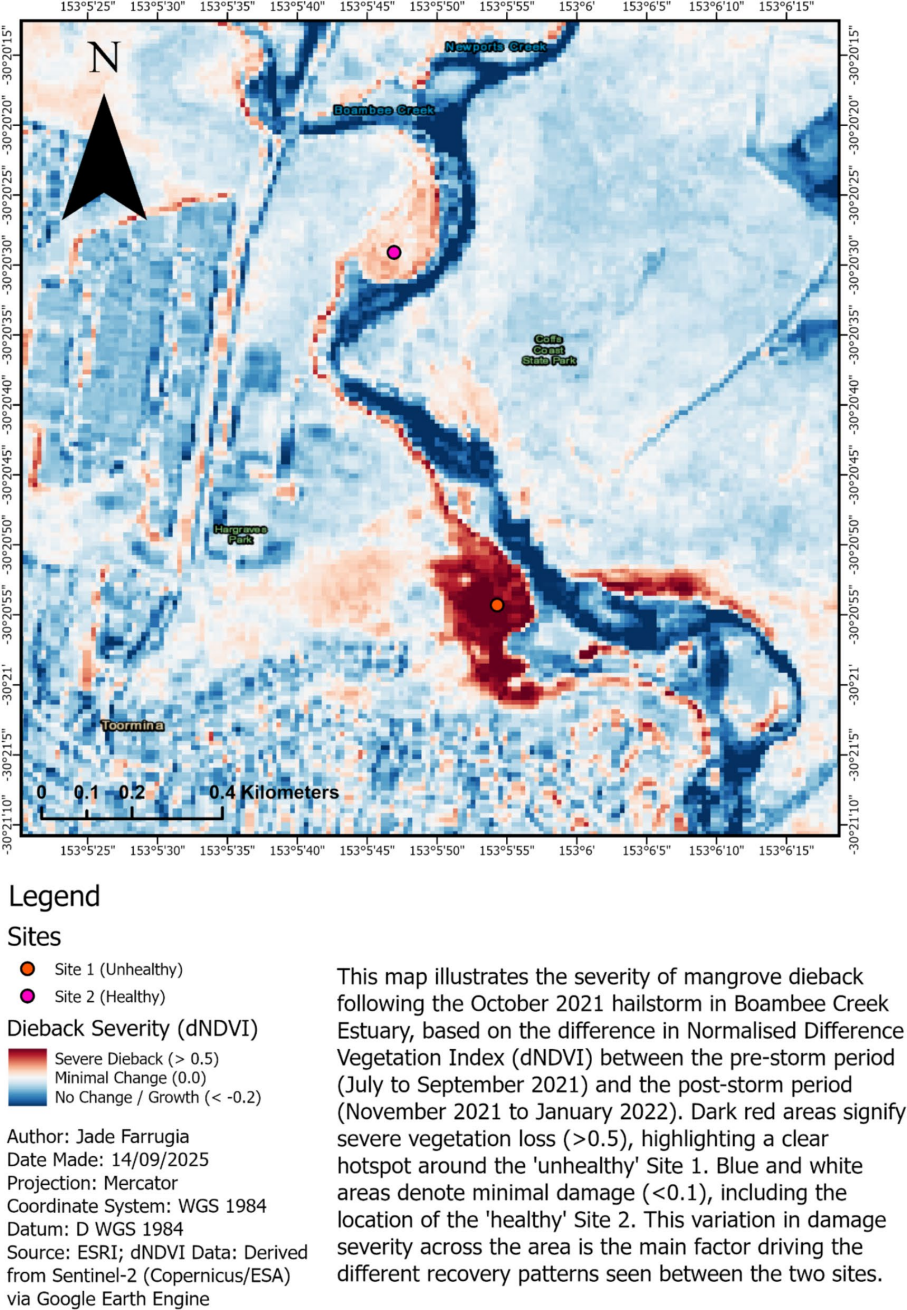
Spatial damage extent map (dNDVI) of the Boambee Creek Estuary. Classification relies on the difference between pre- and post-storm median Sentinel-2 composites. The Dead Zone (Dark Red, dNDVI > 0.5) is spatially coherent and geomorphically confined to the low-elevation basin at site 1. In contrast, the Survivor Zone (blue/white, dNDVI < 0.1) surrounding site 2 indicates minimal canopy loss. This clear spatial boundary confirms that the impact was not diffuse (as expected from chemical drift) but physically and topographically constrained

The spatial analysis confirms that the mortality was not diffuse or random, as might be expected from a volatile chemical plume, but was instead spatially coherent and geomorphically confined. The change detection map (dNDVI) delineated a specific Dead Zone characterised by a mean dNDVI of − 0.51, indicating total loss of photosynthetic biomass. The surrounding Survivor Zones maintained a mean dNDVI of − 0.07 (Fig. 5), representing only minor canopy thinning consistent with recoverable storm damage.

The pixel-level accuracy assessment (*N* = 114) confirmed this zonation with an overall classification accuracy of 93.9%. The confusion matrix showed a sensitivity of 100% for the Impact zone, meaning that every sampled pixel within the geomorphic basin of site 1 was correctly identified as severely damaged. The high specificity (87.5%) of the control zone indicates that, while the survivor site experienced some peripheral stress, it remained a statistically distinct population from the Dead Zone. This precise spatial delineation argues against a gradient-based toxicity model and instead suggests a threshold-based driver of mortality.

### Topographic determinism: the Death Curve

**Fig. 6:**
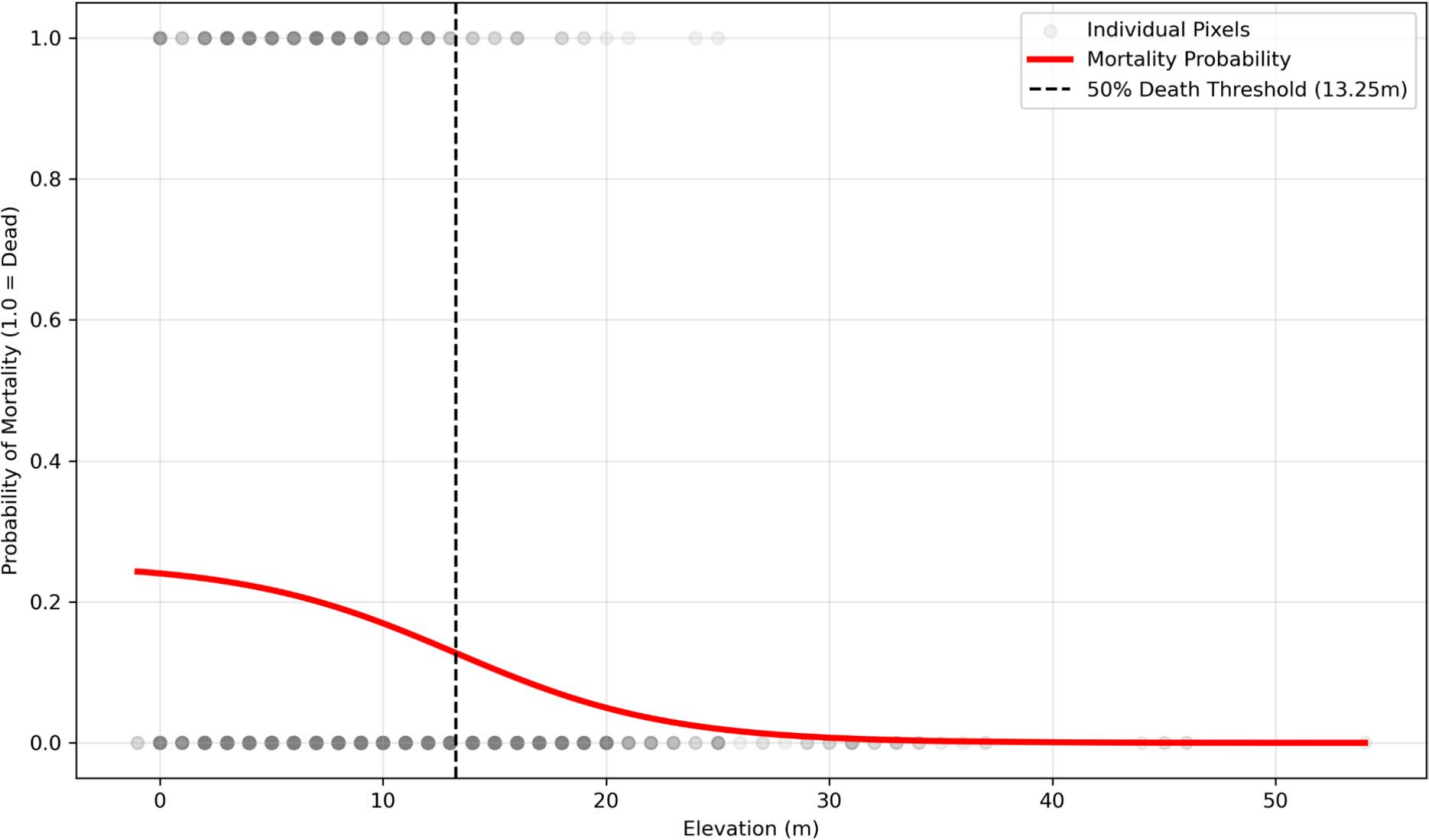
Estuary-wide logistic regression modelling of mortality probability (*N* = 1306). The Death Curve (red line) shows the probability of mangrove mortality as a function of elevation (AHD). The model indicates a strongly non-linear relationship (*p* < 10^–8^), with mortality risk approaching 100% as elevation drops below 1.5 m. The vertical dashed line marks the 50% mortality threshold, confirming that low-elevation basins primarily determined survival

The most decisive outcome of this forensic audit is the identification of elevation as the key variable governing survival. The estuary-wide logistic regression (*N* = 1306) uncovered a strong, non-linear relationship between elevation and mortality. The resulting probability curve (The Death Curve) shows a sigmoid pattern, with the likelihood of mortality approaching 100% as elevation falls below 1.5 m relative to the Australian Height Datum (AHD) (Fig. 6). The statistical significance of this link is undeniable (*p* < 1.6 ✕ 10^–8^), establishing elevation as a universal predictor of survival throughout the entire estuary.

**Fig. 7:**
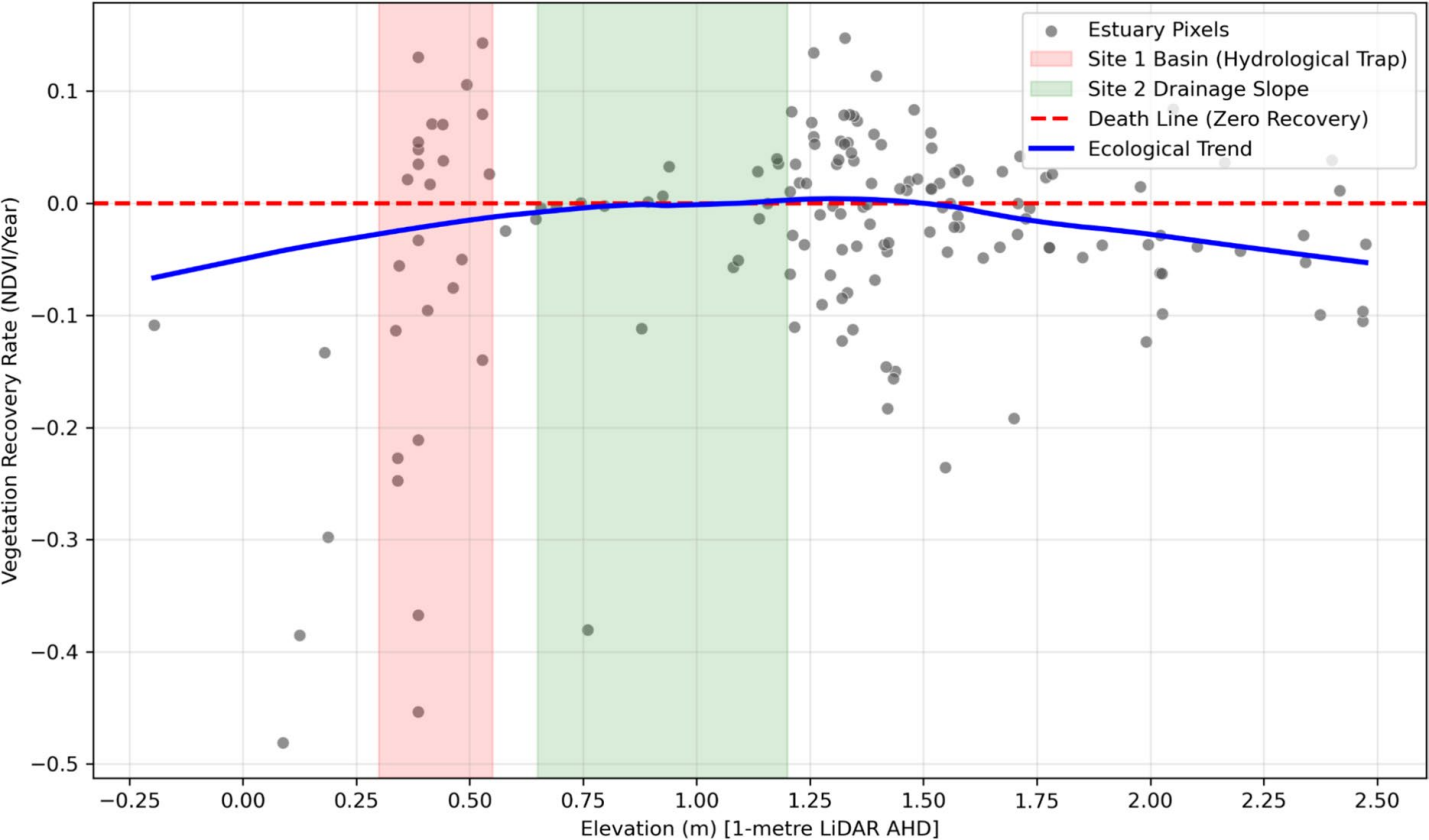
Locally weighted (LOWESS) regression of vegetation recovery rates against elevation, derived from the 1-m LiDAR DEM. The analysis reveals a non-linear topographic control on ecosystem regeneration. The red dashed line marks the Death Line at zero recovery (y = 0). While the immediate riparian fringe (~ 0 m) shows positive recovery due to rapid tidal flushing, recovery plunges into negative decline within stagnant inland depressions (e.g. site 1). Elevated drainage slopes (e.g. site 2) facilitate rapid dewatering and explosive positive recovery

A comparative analysis of the study sites explains the differences in their outcomes. Using the high-resolution 1-m LiDAR DEM, site 1 (The Dead Zone) has a mean elevation of 0.42 m, placing it firmly within the high-risk bathtub zone identified by the logistic model. Site 2 (The Survivor Zone) has a mean elevation of 0.70 m, providing a critical vertical drainage advantage of 0.28 m. Geomorphic slope analysis shows a key difference in drainage potential. Site 1 has a very gentle slope of 1.99°, forming a flat, stagnant basin. Site 2 has a mean slope of 5.09°, which is 2.6 times steeper than the impact site. This topographic setup confirms that site 1 acted as a hydrological sink, holding floodwaters and runoff, while site 2 served as a drainage slope, allowing quick dewatering after the storm surge. This topographic control is further supported by the locally weighted (LOWESS) regression of post-storm recovery rates (Fig. 7), which shows a distinct non-linear relationship. While pixels near 0 m elevation (the immediate riparian fringe) show positive recovery due to rapid tidal flushing into the main channel, the recovery trajectory drops sharply below the survival threshold within the ~ 0.4 m inland basins. It then resumes a positive recovery trend on the higher drainage slopes, confirming that micro-topography dictates ecosystem regeneration.

### The Runoff Paradox

**Fig. 8:**
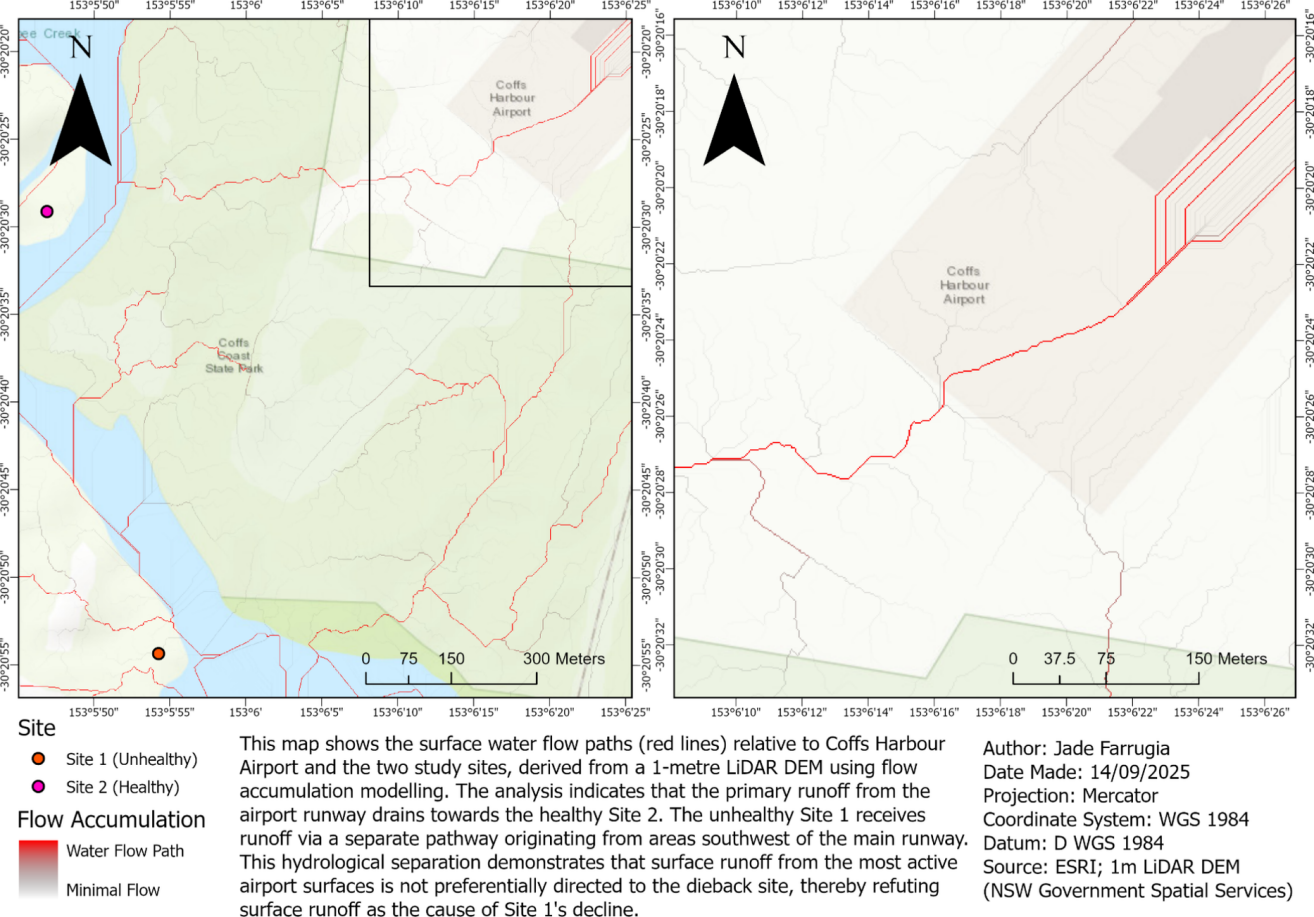
Hydrological flow path modelling of the Boambee Creek Estuary catchment based on a 1-m LiDAR DEM. The analysis shows that the main surface runoff routes (red lines) from the Coffs Harbour Airport runway flow directly into the Survivor Site 2 (pink dot). In contrast, impact site 1 (orange dot) is hydrologically separated from the main runway discharge. This separation questions the idea that runway runoff caused the mortality at site 1

The hydrological routing analysis carried out in ArcGIS Pro challenges the chemical toxicity hypothesis. The flow accumulation modelling shows that the main surface runoff pathways from the Coffs Harbour Airport runway and tarmac surfaces flow directly into the catchment of site 2 (The Survivor) (Fig. 8). Site 1 remains hydrologically separated from direct runway runoff, receiving mainly runoff from the nearby non-industrial forested catchment.

This finding presents a Runoff Paradox for the chemical hypothesis. If the runoff contained lethal levels of hydrocarbons or unburnt fuel, site 2, which directly receives the hydrological flow, should have experienced equal or higher mortality than site 1. Site 2 showed significant resilience and fully recovered. The site most exposed to the supposed pollution source’s runoff empirically indicates that the runoff water quality was sublethal. The mortality at Site 1 cannot be credited to the water source (airport runoff), but must be due to the water’s residence time, which is entirely determined by the basin’s geomorphology.

## Discussion

### The mechanism of mortality: the hydrological trap

The results of this forensic reconstruction offer compelling evidence that the mangrove dieback at Boambee Creek was caused by a biophysical failure best described as a hydrological trap. While the initial trigger was certainly the physical trauma from the 20 October 2021 hailstorm, confirmed by the 4.10σ precipitation Z-Score (Table 1) and the immediate structural break in the Sentinel-2 time series (Fig. 3), the specific morphology of the damage is crucial. The meteorological data confirm a low-wind, high-intensity vertical event (Fig. 2), which explains why the trees were defoliated in situ rather than uprooted. The ongoing dieback at site 1 was entirely determined by basin geomorphology.

The logistic regression analysis establishes a statistically significant connection (*p* < 10^–8^) between elevation and survival, identifying a mortality threshold of approximately 1.5 m AHD (Fig. 6). This finding aligns with the theoretical framework of peat collapse and drowning described in mangrove ecophysiology (Cahoon et al., 2003). Mangroves depend on a delicate balance between hydroperiod and aeration; the aerial root systems must be exposed to the atmosphere for a critical part of the tidal cycle to enable oxygen diffusion to the rhizosphere (Scholander et al., 1955).

The 2.00 m elevation deficit and negligible slope (< 2°) at site 1 characterise it as a topographic depression with limited drainage capacity (Table 1). Following the catastrophic defoliation by hail, the cessation of canopy transpiration, the primary mechanism for pumping water out of the soil profile (Steudle, 2001), combined with the stagnant topography, led to a state of permanent inundation. Similar mechanisms of delayed mortality due to ponding have been observed in Florida following Hurricane Irma (Radabaugh et al., 2020). Lacking the gravitational head to drain (unlike the steeper site 2), the soil at site 1 likely transitioned rapidly to severe anoxia (McKee, 1993). Under such conditions, root respiration ceases, toxic sulphides accumulate and mortality becomes irreversible within weeks, regardless of any chemical contaminants.

This hydrological trap mechanism explains why the dieback was spatially variable despite the uniform delivery of the meteorological shock. The storm did not target site 1 because it was near a flight path; it targeted site 1 because it was the only area where the physical removal of the canopy resulted in a fatal hydrological failure.

### Global parallels: pulse-press interactions in mangrove diebacks

The interaction between an acute pulse disturbance (hail) and a chronic press stressor (poor drainage) observed in Boambee Creek reflects mechanisms found in major mangrove diebacks worldwide. The large 2015 dieback of *Avicennia marina* in the Gulf of Carpentaria, Australia, was linked to a similar combination of climatic stress (El Niño-driven drought) and local hydrological failure, where lower sea levels disconnected the mangroves from their water source (Duke et al., 2017). After Hurricane Irma (2017) in Florida, patchy mortality was associated with ponding in microtopographic depressions where storm surge water could not recede, leading to drowning in a manner very similar to the Boambee Creek event (Radabaugh et al., 2020).

These global case studies reaffirm the validity of the physical-hydrological hypothesis. They show that when the biophysical limits of mangrove resilience are exceeded by a pulse event, the recovery or collapse path is almost always shaped by the local hydrological regime (Fig. 7). The Boambee Creek event adds to this global knowledge by illustrating that hailstorms, often overlooked in favour of cyclones, can generate enough kinetic energy to trigger this peat collapse cascade in vulnerable, low-lying basins (Houston, 1999).

### Surface runoff routing and the compound disturbance framework

While the hydrological flow path modelling (Fig. 8) clearly shows a surface topographic separation, routing the primary airport runway runoff towards the healthy site 2, this does not equate to complete hydrological or groundwater isolation. The topographic basin at Site 1, combined with the extreme > 1-in-100-year hailstorm, served as the acute, primary physical trigger for the sudden canopy collapse. Attributing the lack of subsequent recovery solely to this physical trauma risks oversimplifying a highly complex, multi-stressor estuarine system. It is highly probable that historical or ongoing chemical factors (such as nutrient loading, contamination or altered biogeochemical cycling) operate within a compound disturbance framework. While the acute physical shock of the hailstorm initiated the dieback, underlying chemical stressors may act as compounding factors that inhibit the ecosystem’s long-term physiological resilience and capacity for natural regeneration.

This conclusion is further supported by the survival of the seagrass (*Zostera muelleri*) bio-indicators near the impact zone. Seagrasses are widely regarded as indicators of estuarine water quality because they are highly sensitive to water-column turbidity and dissolved hydrocarbons (Wilson & Ralph, 2012). Their continued survival indicates that the water remained chemically benign throughout the event. The selective death of the mangroves at site 1, therefore, points to mechanisms that affect trees but not submerged grasses: aerial physical trauma and root-zone anoxia.

### Methodological strengths: from anecdote to audit

The main strength of this study is its shift from localised, site-specific field observations to the statistical robustness of landscape-scale remote sensing data. Previous evaluations were limited by selection bias, where the choice of sampling sites influenced the outcomes, a common issue of pseudoreplication (Hurlbert, 1984). By performing a stratified random audit across the entire estuary, this study removed such bias and uncovered landscape-scale laws (e.g. the Death Curve) (Fig. 6) that were hidden in point-based sampling.

The application of a BACI design using high-frequency satellite telemetry enabled precise temporal isolation of the trigger event. While field sampling conducted months after the event can only infer the cause of death from residual evidence (often leading to the Association is not Causation error regarding hydrocarbons), the Sentinel-2 time series provided a real-time physiological trace of the collapse (Fig. 3). This ability to identify the exact week of the state change (*p* < 0.001) was crucial in confirming the hailstorm as the key factor.

### Limitations

Despite the strength of the geospatial approach, this study recognises certain limitations. The spatial resolution of Sentinel-2 (10 m) and the SRTM/LiDAR DEMs (1–30 m) inherently generalise micro-topographic features. While adequate for landscape-scale modelling, sub-metre variations in topography (crab-hole micro-relief) that affect seedling recruitment cannot be resolved. Although the Bathtub Effect hypothesis is strongly supported by the convergence of topographic, spectral and global comparative evidence (Fig. 7), no real-time soil redox potential sensors were active in the basin during the storm. The diagnosis of anoxia is inferred from geomorphic boundary conditions rather than direct measurement. While the LOWESS regression (Fig. 7) accurately captures the catastrophic drowning in low-elevation basins (< 0.5 m), the slight negative trend at the highest elevations (> 1.5 m) should not be conflated with the hydrological trap mechanism. These high-elevation pixels represent the upper intertidal strand zone. Trees in this zone experience infrequent tidal inundation and are subject to chronic hypersalinity and background physiological stress, limiting their capacity for rapid epicormic regeneration after acute defoliation. Although acute chemical toxicity is ruled out as the primary physical trigger of the initial canopy collapse, based on the spatial patterns of survival and surface flow vectors (Fig. 8), this study did not conduct new chemical assays. Remote sensing alone has known limitations for diagnosing subsurface biogeochemical stress. We propose a compound disturbance framework. While the hailstorm acted as the acute physical trigger, historical land use, altered biogeochemical cycling or nutrient loading (Reading et al., 2020) may act as compounding secondary stressors that inhibit long-term recovery (Benkendorff et al., 2025). This highlights the critical need for future in situ testing to supplement remote sensing models.

### Future directions and management implications

The findings of this study indicate a shift in management strategy for Boambee Creek and similar urbanised estuaries. Remediation efforts centred on chemical cleanup or altering flight paths are unlikely to prevent future diebacks, as the cause is geomorphic, not toxicological (Fig. 6). Future research should focus on installing in situ hydrological monitoring stations (piezometers) to measure the hydroperiod and drainage rates in low-lying basins.

This forensic protocol, which combines BACI satellite analysis with hydro-topographic auditing, should be adopted as a standard first-response tool for investigating coastal vegetation diebacks. By quickly ruling out or confirming physical causes, managers can avoid costly and ineffective interventions based on speculative chemical links. For Boambee Creek, the priority must be restoring hydrological connectivity to the site 1 basin, possibly through engineering drainage channels, to prevent water stagnation during future extreme weather events (Fig. 7), a strategy aligned with the principles of Ecological Mangrove Restoration (Lewis, 2005).

## Conclusion

The investigation into the 2021 mangrove dieback at Boambee Creek Estuary demonstrates the critical value of forensic geospatial science in coastal ecology. By scaling up from localised field sampling to a landscape-scale audit, this study recontextualises the mortality event from a purely chemical-toxicity narrative to a compound-disturbance framework. The meteorological reconstruction and real-time Sentinel-2 physiological tracing identified an extreme, > 1-in-100-year hailstorm as the acute physical trigger. The resulting catastrophic defoliation halted canopy transpiration and, when combined with the geomorphic reality of a low-elevation, stagnant basin (a hydrological trap), led to rapid soil anoxia and root drowning. While acute runway runoff toxicity is refuted as the primary trigger, as evidenced by the survival of the forest directly receiving the airport’s hydrological load, historical or groundwater-driven chemical factors may still act as secondary compounding stressors that inhibit recovery. Future coastal management must prioritise restoring hydrological connectivity to vulnerable basins to mitigate drowning risks during extreme weather events, while continuing to integrate high-resolution spatial models with in-situ biogeochemical assays.

## Data Availability

No datasets were generated or analysed during the current study.
